# RNA-Seq analysis of nodule development at five different developmental stages of soybean (*Glycine max*) inoculated with *Bradyrhizobium japonicum* strain 113-2

**DOI:** 10.1038/srep42248

**Published:** 2017-02-07

**Authors:** Song L. Yuan, Rong Li, Hai F. Chen, Chan J. Zhang, Li M. Chen, Qing N. Hao, Shui L. Chen, Zhi H. Shan, Zhong L. Yang, Xiao J. Zhang, De Z. Qiu, Xin A. Zhou

**Affiliations:** 1 Key Laboratory of Oil Crop Biology, Ministry of Agriculture, Wuhan 430062, China; 2 Oil Crops Research Institute of Chinese Academy of Agriculture Sciences, China

## Abstract

Nodule development directly affects nitrogen fixation efficiency during soybean growth. Although abundant genome-based information related to nodule development has been released and some studies have reported the molecular mechanisms that regulate nodule development, information on the way nodule genes operate in nodule development at different developmental stages of soybean is limited. In this report, notably different nodulation phenotypes in soybean roots inoculated with *Bradyrhizobium japonicum* strain 113-2 at five developmental stages (branching stage, flowering stage, fruiting stage, pod stage and harvest stage) were shown, and the expression of nodule genes at these five stages was assessed quantitatively using RNA-Seq. Ten comparisons were made between these developmental periods, and their differentially expressed genes were analysed. Some important genes were identified, primarily encoding symbiotic nitrogen fixation-related proteins, cysteine proteases, cystatins and cysteine-rich proteins, as well as proteins involving plant-pathogen interactions. There were no significant shifts in the distribution of most GO functional annotation terms and KEGG pathway enrichment terms between these five development stages. A cystatin Glyma18g12240 was firstly identified from our RNA-seq, and was likely to promote nodulation and delay nodule senescence. This study provides molecular material for further investigations into the mechanisms of nitrogen fixation at different soybean developmental stages.

Legumes interact with specific soil rhizobia to develop symbiotic relationships that lead to the formation of root nodules. These nitrogen-fixing nodules allow the host plants to grow without the addition of nitrogen fertilizers and are of significant agronomic and ecological importance. Nodule development is initiated by an exchange of chemical signals between legumes and soil rhizobia and is accompanied by a series of signal transductions inside the legume’s root cells^1^,^2^. The early molecular events involved in nodule initiation are quite well understood^3^,^4^,^5^,^6^. Although some legume genes have been implicated in the late developmental stage of nodule development and/or bacteroid differentiation^7^,^8^,^9^,^10^,^11^,^12^ and some studies have investigated the molecular mechanisms that regulate nodule development^13^,^14^, information on nodule-related genes in the middle or late developmental stage of nodule development is also limited.

Soybean (*Glycine max*), which had a global harvest area in 2015 of more than 1,734 million acres, is an important oil crop, food and feed material, and requires a large amount of nitrogen for growth. However, excessive application of nitrogen fertilizer not only reduces the effective nitrogen utilization of soybean (nitrogen uptake, utilization and fixation efficiencies), but also results in reduced production efficiency, a waste of resources, environmental pollution and other issues^15^,^16^. Studies on how to reduce the amount of nitrogen fertilizer and increase crop nitrogen utilization efficiency are very important, and there have been some good studies on the high nitrogen utilization efficiency of soybean^17^,^18^,^19^. For symbiotic nitrogen fixation, the premature senescence of nodules can negatively affect nitrogen availability for soybean growth; thus, the development and senescence of nodules require further detailed study^11^. Nodule development directly affects nitrogen fixation efficiency, which changes with the growth of soybean^20^. A soybean developmental stage classification based on significant changes in external morphological characteristics has been in place since 1971^21^ and plays important roles in soybean cultivation and fertilizer application^10^; the characteristics of nitrogen fixation at different developmental stages are critical in agriculture and ecology. However, little is known about the molecular mechanisms regulating nodule development and/or nitrogen fixation at different developmental stages.

Knowledge of the sequence information related to nodule development in soybean was greatly enhanced by the release of high-quality drafts of the *G. max, M. truncatula* and *Phaseolus vulgaris* genomes^6^,^22^,^23^. Moreover, a huge EST collection of more than 390,000 sequences in soybean has been released (http://www.ncbi.nlm.nih.gov/dbEST/) together with approximately 40,000 full-length cDNA clones^24^. A comparative analysis of genome sequences in six legumes revealed many of the symbiotic genes in soybean^25^, and RNA-Seq transcription data predicted several nodulation-related gene regulatory networks^26^. However, this genome-based information is constrained by prior knowledge of gene sequences and limits the patterns of gene expression at various stages of nodule development. We are in a position to significantly improve our understanding of soybean nodule development using RNA-Seq, which is an effective method that produces quantitative data related to transcripts with greater sensitivity, higher reproducibility, and wider dynamic range^27^ than other conventional methods. This method also has relatively little variation between technical replicates for identifying differentially expressed genes^28^.

In this report, we investigated ten comparisons between five important developmental stages (branching stage, flowering stage, fruiting stage, pod stage and harvest stage) of soybean and identified a large number of differentially expressed genes (DEGs), including those encoding soybean symbiotic nitrogen fixation-related proteins, cysteine proteases, cystatins and cysteine-rich proteins, as well as proteins involved in plant-pathogen interactions. Seven cystatins are actively transcribed during nodule development and senescence^11^, while two other cystatins (Glyma18g12240 and Glyma18g00690) were identified from our RNA-seq. The symbiotic function of Glyma18g12240 was studied to verify the reliability of our data, and the results showed that it was likely to promote nodulation and delay nodule senescence. The discovered differentially expressed genes (DEGs) and the results described in this study should aid efforts to understand the mechanisms of soybean nodule development and shed new light on the molecular mechanisms of nitrogen fixation at five important developmental stages.

## Results

### Nodulation phenotypic characterization at different developmental periods of soybean

The nitrogen fixation rate of soybean nodules changes with plant growth. The nodules begin to fix nitrogen when leghemoglobin (*Lb*) is expressed (branching stage); the nitrogen fixation rate then gradually increases, is relatively high at the flowering and fruiting stages and then gradually weakens from the pod stage; and at the harvest stage, the nodules are senescence and almost incapable of nitrogen fixation^29^,^30^.

**Figure 1:**
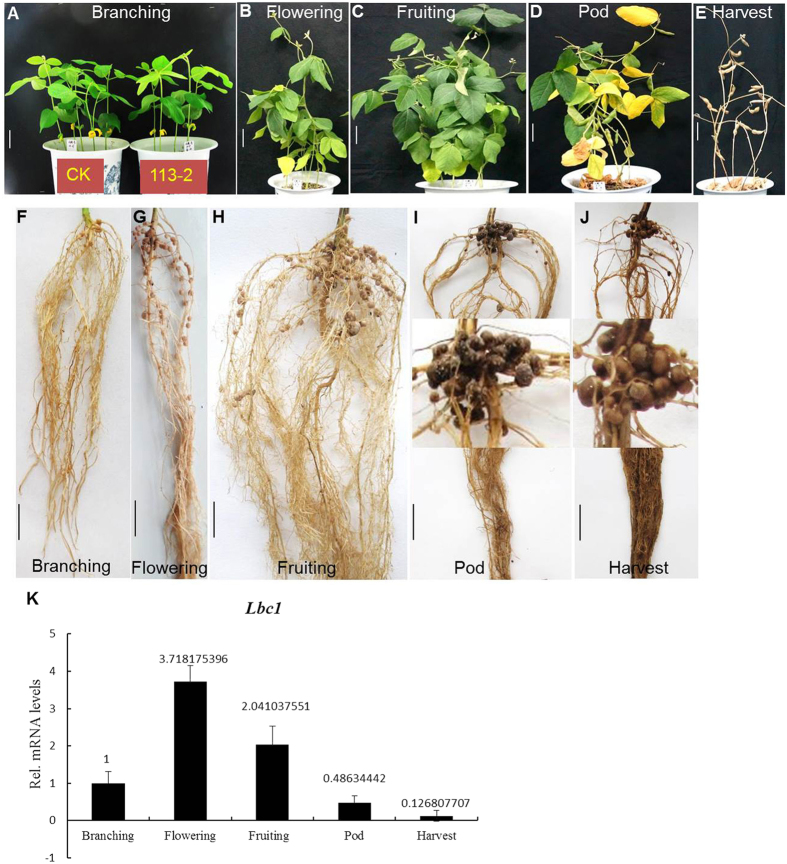
Symbiotic phenotype features of five important soybean developmental stages. **(A–E)** Soybean growth at five important developmental stages, including the branching stage, flowering stage, fruiting stage, pod stage and harvest stage. (**F–J)** Nodulation phenotypes were examined at five developmental stages after inoculation with 113-2. **(K**) qPCR analysis of the transcript levels of *Lbc1* (Glyma10g34280) at five developmental stages. Bars, 4 cm (**A,B**); 5 cm (**C–E**); 3 cm (**F–J**). d, days.

To investigate nodule development during soybean growth, we examined the symbiotic phenotypic of soybean inoculated with *B. japonicum* 113-2 at five developmental stages (branching stage, flowering stage, fruiting stage, pod stage and harvest stage), and the results are shown in Fig. 1 and Table S1. The classification of these five developmental stages according to the popular classification system^21^ and the detail defined information for the plants at each stage was shown in materials and methods. The growth of soybean inoculated with *B. japonicum* 113-2 at these five developmental stages was shown in Fig. 1A–E. At the branching stage, the control (CK, inoculated with media only) had slightly better growth than the soybean inoculated with *B. japonicum* 113-2 (Fig. 1A), maybe because the symbiotic process required more energy from the host plant and nitrogen fixation was weak during this period. The number of nodules per plant and the dry weight per nodule increased from the branching stage to fruiting stage and decreased from the pod stage (Fig. 1F–J, Table S1). At the branching stage, flowering stage and fruiting stage, the nodules were pink and/or yellow, while at the pod stage, the nodules became brown (Fig. 1G–I). The nodules were senescence and filled with water at the harvest stage (Fig. 1I,J). Moreover, differentially expressed levels of *Gm Lb (Glyma10g34280*), which is required for nitrogen fixation^31^,^32^, at all five developmental stages were shown (Fig. 1K). These results indicate notable symbiotic phenotypes among different developmental stages of soybean.

### Quality assessment of RNA-Seq and DEG identification in different developmental periods of soybean

RNA-Seq was performed for soybean nodule samples at five developmental stages of soybean, and detailed information is shown in the materials and methods. The proportion of clean reads among the total acquired reads was more than 99.6%, indicating high-quality sequencing (Fig. S1). Comparable numbers of total mapped reads, perfect match reads and unique match reads were obtained in the mapping results for the five developmental stages (Table S2). Sequencing saturation analysis showed that the number of genes represented by clean reads stabilized when the number of total tags reached 2.5 million or higher; therefore, the obtained reads represented full coverage for each sample (Fig. S2). During the RNA-Seq experiment, mRNAs are first broken into short segments by chemical methods and then sequenced. The results showed that the distribution of reads on reference genes was sufficient for subsequent bioinformatics analysis (Fig. S3). These results indicate that the sequencing library was of good quality and contained sufficient information for gene expression analysis.

**Figure 2:**
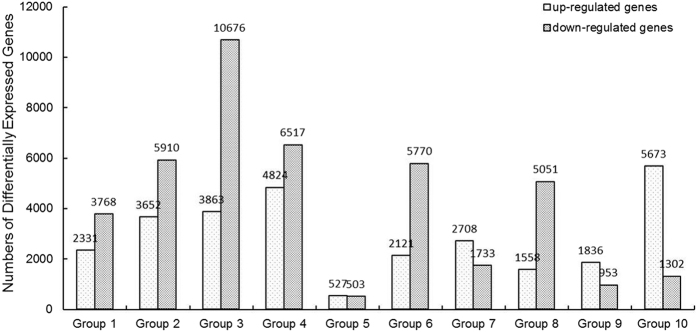
Differentially expressed genes (DEGs) between different developmental periods of soybean. Group 1: branching stage vs. flowering stage; Group 2: branching stage vs. fruiting stage; Group 3: branching stage vs. pod stage; Group 4: branching stage vs. harvest stage; Group 5: flowering stage vs. fruiting stage; Group 6: flowering stage vs. pod stage; Group 7: flowering stage vs. harvest stage; Group 8: fruiting stage vs. pod stage; Group 9: fruiting stage vs. harvest stage; Group 10: pod stage vs. harvest stage.

To judge the significance of differences in DEGs between five developmental stages of soybean, a false discovery rate (FDR) ≤0.001 and |log2 ratio| ≥1 were used as criteria for the ten Groups (detailed information is provided in the materials and methods). Information on the DEGs in the ten Groups between the five nodule samples is provided in Table S3, and the numbers of up-regulated and down-regulated DEGs in these ten Groups are shown in Fig. 2. These included 6,099 DEGs (2331 up-regulated, 3768 down-regulated) between the branching stage and flowering stage; 9,562 DEGs (3652 up-regulated, 5910 down-regulated) between the branching stage and fruiting stage; 14,539 DEGs (3863 up-regulated, 10676 down-regulated) between the branching stage and pod stage; 11,341 DEGs (4824 up-regulated, 6517 down-regulated) between the branching stage and harvest stage; 1,030 DEGs (527 up-regulated, 503 down-regulated) between the flowering stage and fruiting stage; 7,891 DEGs (2121 up-regulated, 5770 down-regulated) between the flowering stage and pod stage; 4,441 DEGs (2708 up-regulated, 1733 down-regulated) between the flowering stage and harvest stage; 6,609 DEGs (1558 up-regulated, 5051 down-regulated) between the fruiting stage and pod stage; 2,789 DEGs (1836 up-regulated, 953 down-regulated) between the fruiting stage and harvest stage and 6,975 DEGs (5673 up-regulated, 1302 down-regulated) between the pod stage and harvest stage.

### Functional ontology and KEGG pathway enrichment analysis of DEGs

**Figure 3:**
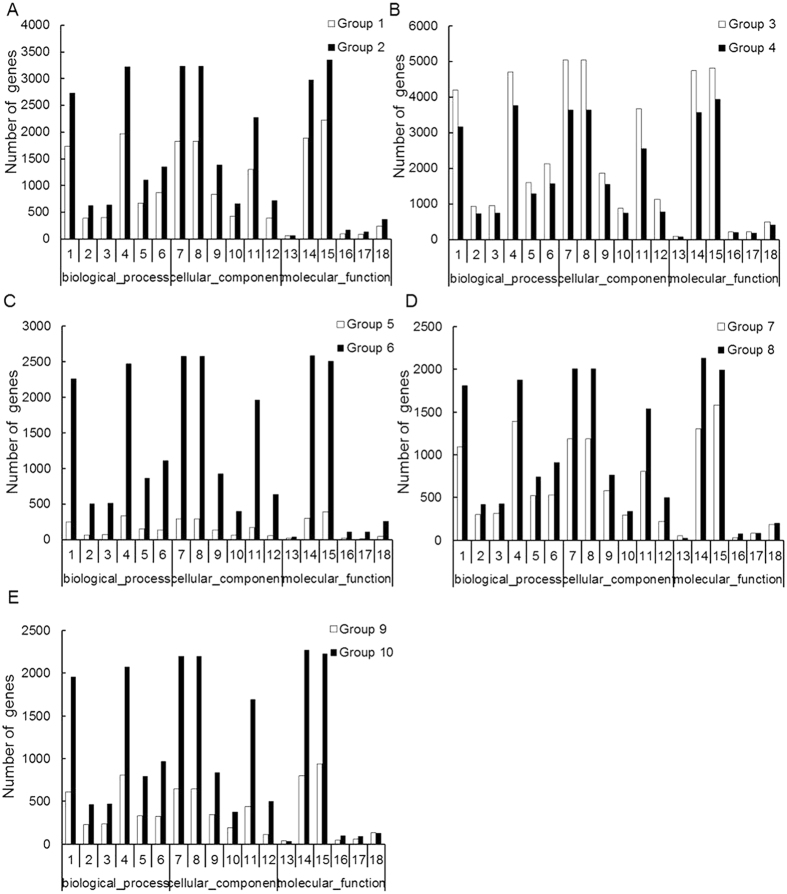
Gene Ontology - based functional annotation of DEGs between different developmental periods of soybean. The three GO domains - biological process, cellular components, and molecular function - are shown. The numbers of genes in each term are shown in histograms. Eighteen GO function terms are indicated: 1, metabolic process; 2, cellular process; 3, response to stimulus; 4, single-organism process; 5, establishment of localization; 6, localization; 7, cell; 8, cell part; 9, membrane; 10, organelle; 11, membrane part; 12, organelle part; 13, catalytic activity; 14, binding; 15, transporter activity; 16, nucleic acid binding transcription factor activity; 17, molecular transducer activity; 18, antioxidant activity. **(A)** Group 1 (branching stage vs. flowering stage) and Group 2 (branching stage vs. fruiting stage). **(B)** Group 3 (branching stage vs. pod stage) and Group 4 (branching stage vs. harvest stage). **(C)** Group 5 (flowering stage vs. fruiting stage) and Group 6 (flowering stage vs. pod stage). **(D)** Group 7 (flowering stage vs. harvest stage) and Group 8 (fruiting stage vs. pod stage). **(E)** Group 9 (fruiting stage vs. harvest stage) and Group 10 (pod stage vs. harvest stage).

An internationally standardized gene function classification system, Gene Ontology, was used to classify the DEGs in the above-mentioned 10 Groups. A total of 18 gene ontology function terms are listed in Fig. 3 and divided into three categories: biological process, cellular components, and molecular function. For all 10 Groups, the biological processes associated with the DEGs mainly focused on metabolic process, cellular process, response to stimulus, single-organism process, establishment of localization and localization. The cellular components mainly included cell, cell part, membrane, organelle, membrane part and organelle part. The main molecular functions of the DEGs were catalytic activity, binding, transporter activity, nucleic acid binding transcription factor activity, molecular transducer activity and antioxidant activity (Fig. 3).

**Figure 4:**
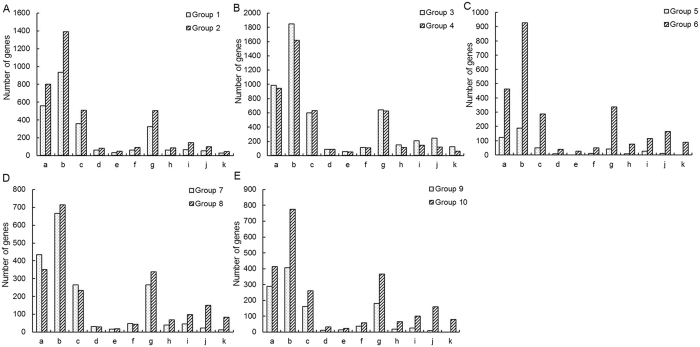
Results of KEGG pathway enrichment analyses of DEGs for 11 KEGG pathways. The x- and y-axes represent pathway categories and the number of genes in each pathway, respectively. a, Biosynthesis of secondary metabolites; b, Metabolic pathways; c, Plant hormone signal transduction; d, Cysteine and methionine metabolism; e, Nitrogen metabolism; f, ABC transporters; g, Plant-pathogen interaction; h, Ubiquitin mediated proteolysis; i, Protein processing in endoplasmic reticulum; j, RNA transport; k, mRNA surveillance pathway. **(A)** Group 1 (branching stage vs. flowering stage) and Group 2 (branching stage vs. fruiting stage). **(B)** Group 3 (branching stage vs. pod stage) and Group 4 (branching stage vs. harvest stage). **(C)** Group 5 (flowering stage vs. fruiting stage) and Group 6 (flowering stage vs. pod stage). **(D)** Group 7 (flowering stage vs. harvest stage) and Group 8 (fruiting stage vs. pod stage). **(E)** Group 9 (fruiting stage vs. harvest stage) and Group 10 (pod stage vs. harvest stage).

KEGG is the major public database for pathway enrichment analysis^33^ and the eleven KEGG pathway subgroups associated with the DEGs are shown in Fig. 4. The pathways with the greatest numbers in each group were metabolic pathways, and other pathways, such as biosynthesis of secondary metabolites, plant hormone signal transduction and plant-pathogen interaction, were also main enriched pathways.

GO functional annotation and a pathway enrichment analysis of DEGs in soybean nodule development revealed no high shift in the distribution of most terms in the ten Groups.

### DEGs associated with plant-pathogen interactions

A legume-derived flavonoid can induce the pathogenic type III secretion system (T3SS) of rhizobia, which injects effector proteins into host cells to modulate nodulation signalling towards nodulation and to abort the nodulation process through recognition by the host defence system^34^,^35^.

**Figure 5:**
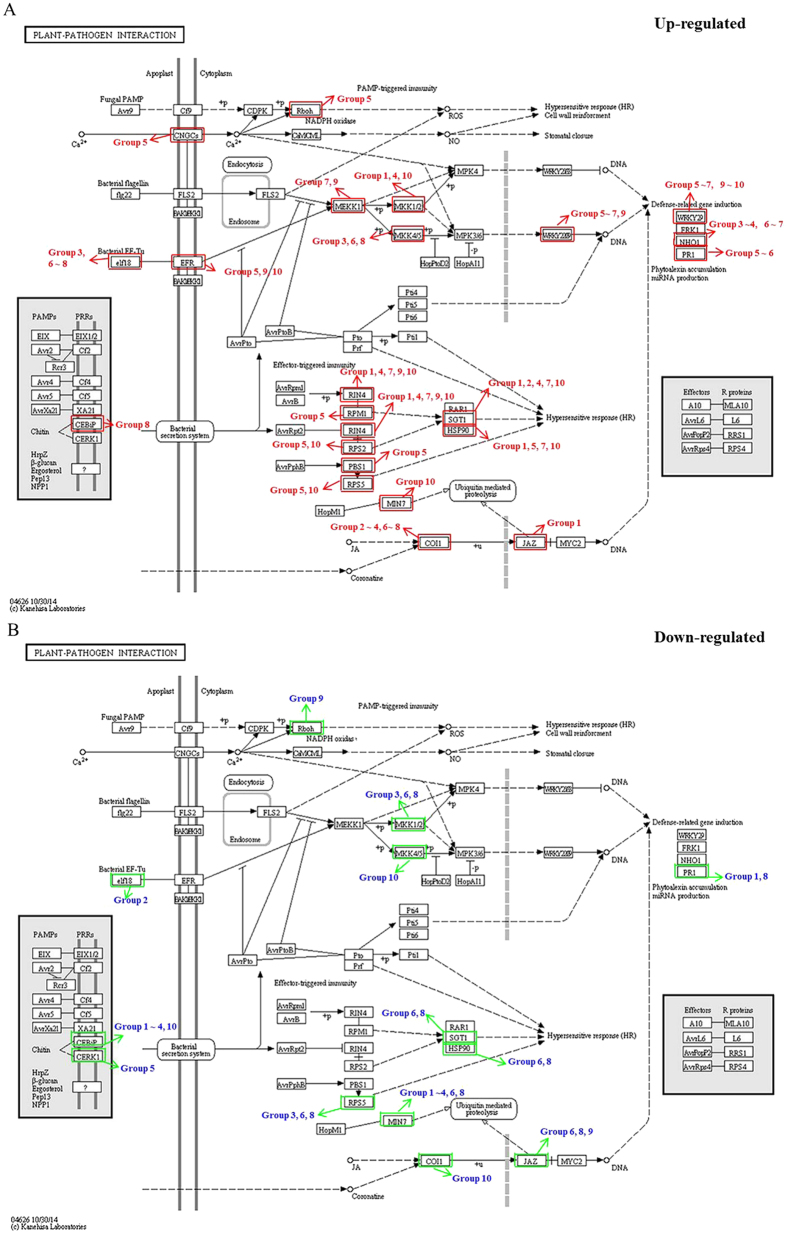
DEGs associated with the plant-pathogen interaction pathways between different developmental periods of soybean. **(A)** Up-regulated genes are boxed in red, and the red arrows point out the up-regulation of DEGs in Groups of nodule development. **(B)** Down-regulated genes are boxed in green, and the green arrows point out the down-regulation of DEGs in Groups. Group 1: branching stage vs. flowering stage; Group 2: branching stage vs. fruiting stage; Group 3: branching stage vs. pod stage; Group 4: branching stage vs. harvest stage; Group 5: flowering stage vs. fruiting stage; Group 6: flowering stage vs. pod stage; Group 7: flowering stage vs. harvest stage; Group 8: fruiting stage vs. pod stage; Group 9: fruiting stage vs. harvest stage; Group 10: pod stage vs. harvest stage.

To study the regulation of the soybean-soil rhizobia interaction during nodule development, we analysed the plant-pathogen interaction KEGG pathway (obtained by KEGG, http://www.kegg.jp/kegg/kegg1.html) in more detail (Fig. 5). A total of 24 selected KEGG gene sets in this pathway were differentially expressed between different developmental periods of soybean. One gene that matched this pathway (MIN7^36^) was only up-regulated in Group 10 and down-regulated in the other Groups, while the other two genes, MAPK kinase (MKK4/5)^37^ and coronatine-insensitive protein 1 (COI1)^38^, were down-regulated in Group 10 and up-regulated in the other Groups. Jasmonate ZIM domain-containing protein (JAZ)^39^ was up-regulated in Group 1 and down-regulated in Groups 6, 8 and 9. Elongation factor Tu 18 (elf18)^40^ was down-regulated in Group 2 and up-regulated in Groups 3, 6, 7 and 8. Pathogenesis-related protein 1 (PR1)^41^ was down-regulated in Group 1 and up-regulated in Groups 5, 6 and 8, and chitin elicitor-binding protein (CEBiP)^42^ was down-regulated in Group 8 and up-regulated in Groups 1–4 and 10. The DEGs in these gene sets with ≥8-fold changes are listed in Table S4; some of these DEGs existed in two or more Groups, for example, *Glyma18g51546* and *Glyma16g34030* were in Groups 6, 8 and 10, and so on. These results indicate that differential defence responses in soybean nodules are related to nodule development and senescence, and this also uncovers some important genes in the tightly regulated soybean symbiotic process that coordinates nodule development with host soybean immunity defence.

### DEG-encoding cysteine proteases, cystatins and cysteine-rich proteins in nodule development and senescence

Cysteine proteases and cystatins play roles in nodule development; eighteen cysteine proteases and seven cystatins are actively transcribed during nodule development and senescence^11^. Among them, ten cysteine proteases and four cystatins were differentially expressed in nodule development and senescence. Two other cysteine proteases (Glyma04g36470 and Glyma17g18440) and two cystatins (Glyma18g12240 and Glyma18g00690) were also identified from the DEGs (Table S5). Additionally, 44 nodule cysteine rich proteins, which included 33 receptor-like protein kinases, nine secretory proteins and two polycomb-like proteins, were identified from the DEGs associated with nodule development by RNA-Seq, and some of the genes encoded the same protein (Table S5). These proteins were characterized by a putative signal peptide and conserved cysteine residues; however, there has been very limited research into the functions of these cysteine-related genes.

### Analysis of selected symbiotic nitrogen fixation - related genes

Fifty-two soybean genes responsible for the broad NF signal pathway and nodulation were identified by searching for homologues of *M. truncatula* and *L. japonicus* nitrogen fixation-related genes in soybean genome sequence data^22^,^25^,^43^, and most of these orthologues in soybean have been identified in previous works^44^,^45^. Among them, 21 genes were identified as DEGs in this report and their potential symbiotic functions were shown in Table S6. Besides, four important marker genes known to be important in symbiotic nitrogen fixation (ApyraseGS52, Calmodulin-like protein, Enod40 and Nodulin 35)^46^ were examined here to see how their expression changes throughout nodule development and senescence (Table S6).

### Verification of RNA-Seq results by qPCR and functional analysis of the candidate gene *Glyma18g12240*

**Figure 6:**
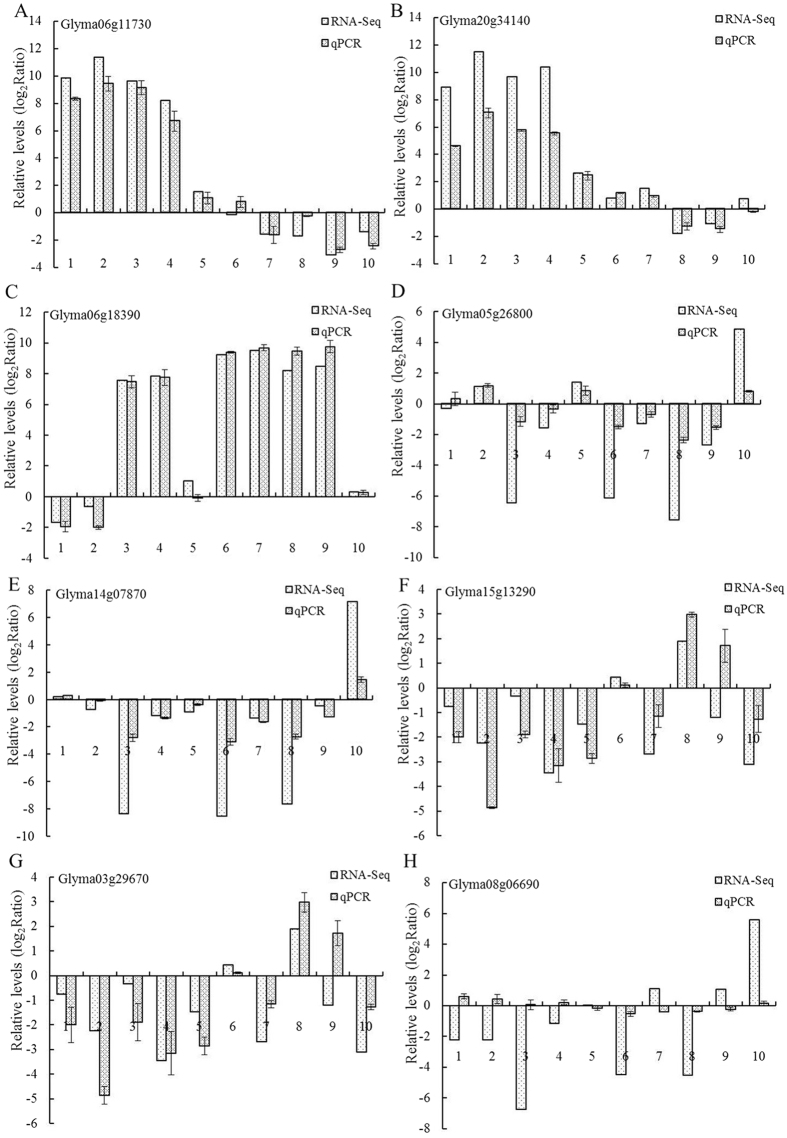
Comparison of expression rates determined by RNA-Seq and qPCR on 8 genes in soybean nodules. Three biological replica samples were used, and all qPCR reactions were repeated three times and the data are presented as the mean ± SD. **(A**) Glyma06g11730. (**B)** Glyma20g34140. (**C)** Glyma06g18390. (**D)** Glyma05g26800. **(E)** Glyma14g07870. (**F)** Glyma15g13290. (**G)** Glyma03g29670. (**H)** Glyma08g06690. QACT was the reference gene for these qPCR experiments.

To verify the RNA-Seq results, first, the expression stabilities of four references genes were evaluated. QACT (GmACT11, Glyma18g52780), Ubiquitin and Eukaryotic elongation factor 1-beta (ELF1B) were ranked the most stable in all of the samples in our experiment, while Glucose-6-phosphate Dehydrogenase (GmG6PD) was consistently ranked poorly (Fig. S4). QACT was selected for qPCR. Eight DEGs that were randomly selected based on the transcriptional profile analysis were measured for each stage. The qPCR results agreed with the transcriptional profile data for 68 out of 80 (85%) data points (Fig. 6). Although the fold changes were not exactly identical, both methods yielded identical expression trends for most of the data points. The qPCR results ultimately reflected consistency with the RNA-Seq data. The sequences of the specific primers that were used for qPCR are given in Table S7.

**Figure 7:**
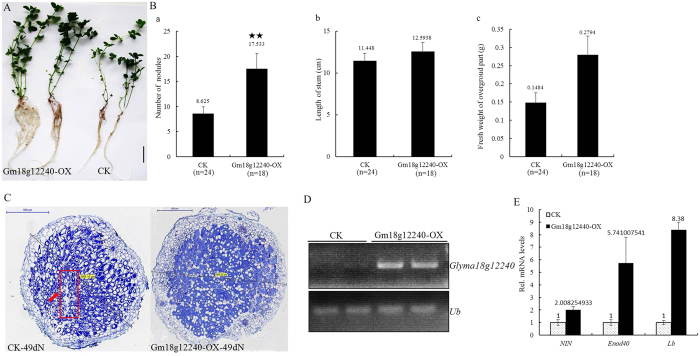
Effect of *Glyma18g12240* overexpression on symbiosis in *L. japonicus*. **(A)** Symbiotic phenotypes of transgenic plants 49 days after inoculation with *M. loti*. MAFF303099. Hairy roots expressing vector pU1301 served as controls. Two independent transgenic plants were chosen for Photography. Bars = 5 mm. **(B)** Mean numbers of nodules per plant, lengths of stem per plant and fresh weights of the aboveground tissues per plant with a standard deviation (SD) of *L. japonicus* expressing pMUb: Glyma18g12240 (*Gm18g12240-OX*) or the empty vector pU1301 (CK) 49 days after inoculation with *M. loti*. The numbers of nodules in the scored plants is indicated in parentheses; “★★” indicates a significant difference between them. P-Values, 0.026 (a); 0.362 (b); 0.184 (c). **(C)** Paraffin-embedded slides stained with toluidine blue in the control and *Gm18g12240-OX* 49 day post-inoculation (dpi) nodules. The lengths of the two nodules were measured by Pannoramic viewer. The red box and the red arrow indicate a small senescent zone in the control 49 dpi nodules. **(D)** Semi-quantitative RT-PCR analysis of the transcript levels of *Glyma18g12240* in the control and *Gm18g12240-OX* hairy roots. **(E)** qPCR analysis of the transcript levels of *NIN, Enod40* and *Lb* in the control and *Gm18g12240-OX* hairy roots. Total RNA isolated from the root system was used for qPCR. Relative expression levels of *NIN, Enod40* and *Lb* transcripts in *Gm18g12240-OX* hairy roots were calculated with reference to those of the control hairy roots.

As described above, two cystatins (Glyma18g12240 and Glyma18g00690) were firstly identified from our RNA-seq (Table S5) and not in the seven cystatins^11^. To verify the reliability of our data, *Glyma18g12240* was expressed under the control of the maize (*Zea mays*) ubiquitin promoter (*Glyma18g12240-OX)* in transgenic hairy roots of *L. japonicus*^47^,^48^. The nodulation phenotypes were scored 49 days after inoculation with *M. loti* MAFF303099, which expresses β-galactosidase (lacZ) as a constitutive marker for the presence of rhizobial cells^49^. Significantly better growth and more nodules were produced in *Glyma18g12240-OX* hairy roots than in the control (Fig. 7A,B). The nodule numbers per root system increased from 8.625 in the control to 17.533 in *Glyma18g12240-OX* hairy roots (Fig. 7Ba), the stem length per plant increased from 11.448 cm in the control to 12.5938 cm in *Glyma18g12240-OX* (Fig. 7Bb), and the fresh weight of the aboveground plant tissues increased from 0.1484 g in the control to 0.2794 g in *Glyma18g12240-OX* (Fig. 7Bc). Paraffin-embedded slides analysis was used to investigate the role of *Glyma18g12240* in nodule senescence; we harvested both control and *Glyma18g12240-OX* nodules at 49 dpi (days post inoculation) and used light microscopy to examine sections that had been stained using toluidine blue. Ageing became evident in control nodules at 49 dpi, while most of the *Glyma18g12240-OX* nodules did not appear to age (Fig. 7C), indicating a delay in nodule senescence in the *Glyma18g12240-OX* nodules. Semi-quantitative RT-PCR results showed that the expression level of *Glyma18g12240* was abundant in *Glyma18g12240-OX* hairy roots (Fig. 7D). The expression levels of two early nodulin genes (*NIN* and *ENOD40*)^50^,^51^ and the expression of *Lb* (leghemoglobin), a typical nodulin gene required for nitrogen fixation^31^, were examined by qPCR, and the results showed that the expression levels of these three nodulin genes increased in *Glyma18g12240-OX* hairy roots compared with those in the control hairy roots, especially for *ENOD40* and *Lb* (Fig. 7E). These results suggest that *Glyma18g12240* promotes nodulation in *L. japonicus* and performs an essential role in delaying nodule senescence. The exact regulation mechanism of *Glyma18g12240* is important and worthy of exploration in further studies.

## Discussion

In this study, RNA-Seq was used to investigate the expression of nodule genes in nodules of soybean inoculated with *B. japonicum* 113-2 at five developmental stages (branching stage, flowering stage, fruiting stage, pod stage and harvest stage). RNA-Seq is an effective method that produces quantitative data related to transcripts with a greater sensitivity, higher repeatability, and wider dynamic range than conventional methods^27^. This method has also been shown to have relatively little variation between technical replicates for identifying DEGs^28^. Consistent with the previous reports, our qPCR results agree with the transcriptional profile data for 68 out of 80 (85%) data points (Fig. 6), suggesting that our RNA-Seq data are reliable.

The high efficiency of nitrogen fixation in soybean developmental stages is critical in agriculture and ecology^20^. However, little is known about the molecular mechanisms regulating nitrogen fixation at different developmental stages, and there has been little research regarding the way nodule genes operate in the middle and/or later stages of nodule development, which directly affect nitrogen-fixation efficiency^11^. To identify the genes controlling the middle and/or later stages of nodule development and elucidate the molecular mechanisms for nitrogen fixation efficiency during soybean development, we focused on five important developmental stages of soybean and identified a large number of nodule-related DEGs from RNA-Seq data. Our results first identified nodule development-related genes from soybean nodules at five important developmental stages of soybean, including those encoding soybean symbiotic nitrogen fixation-related proteins, cysteine proteases, cystatins and cysteine-rich proteins, as well as proteins involved in plant-pathogen interactions. The DEGs that were discovered in this study represent a molecular resource to aid our understanding of the mechanisms of soybean nodule development and nitrogen fixation at different soybean developmental stages.

Nodule development is a complex process that is tightly regulated in the host plant cell, and there has been very limited research on its molecular mechanisms^7^. A total of 1,973 soybean genes were identified from the large-scale transcriptome analysis data generated for soybean root hair cells after rhizobium infection^52^. Several nodulation-related gene regulatory networks were predicted from the RNA-Seq transcription data generated for soybean root hair cells at three different developmental stages (12 h, 24 h, and 48 h) of nodulation after rhizobium infection^26^. A total of 2915 genes were identified as being differentially expressed during the early stages of nodulation from a total mRNA profile using RNA-Seq to target the soybean root tissues responding to compatible rhizobia^5^. From the soybean root subtractive library (non-inoculated × inoculated), 3,210 differentially expressed transcripts were identified, produced by combining the suppressive subtractive hybridization (SSH) technique with Illumina sequencing^44^. A large number of genes were found to be differentially expressed in soybean roots during the late nodule development stage, when active nitrogen fixation occurred^7^. However, these genes were all identified from soybean roots or root hairs and were related mainly to early nodule development. In this report, RNA samples were collected from soybean nodules at five important developmental stages, and related to middle and later nodule development and/or the entire process of nodule nitrogen fixation.

The development of soybean nodules involves a complex series of physiological processes, as indicated by the large numbers of DEGs between five important developmental stages (Fig. 3). Accordingly, GO functional annotation and a pathway enrichment analysis of DEGs revealed few functional differences between the five important developmental stages (Figs 3 and 4). Differences between these stages became much clearer in terms of changes in gene expression (Fig. 2). Group 5 showed the lowest total number of DEGs (Fig. 2), which suggested that nitrogen fixation from the flowering stage to the fruiting stage is relatively stable. Groups 1 to 4, 6 and 8 included more down-regulated DEGs than up-regulated DEGs; while in the Group 10, a general up-regulation of most genes was observed. Interestingly, the up/down-based regulation of DEGs in group 8 (fruiting stage vs. pod stage) was clearly opposite to that observed in group 10 (pod stage vs. harvest stage), indicating that the pod stage is a key turning point for soybean nodule development or symbiotic nitrogen fixation and the initiation of a series of new processes from the pod stage to the harvest stage of soybean, which might be associated with nodule senescence.

Rhizobia can adopt a pathogenic system that stimulates their legume hosts to initiate symbiotic programmes^34^, and the local immune suppression in host legumes induced by rhizobia is essential for the establishment of symbiosis^53^. In addition to NFs, T3SS has been reported to affect symbiosis with host legumes^34^ and is known as an introducer of virulence factors from plant pathogens^54^. The rhizobia T3SS can inject effector proteins into host cells to modulate nodulation signalling towards nodulation and abort the nodulation process by the host legume defence system^34^. Other microbe/pathogen-associated molecular pattern (M/PAMP), such as chitin (Chemical compound), an NF analogue, can induce the M/PAMP-triggered susceptibility (M/PTS) of host legumes to rhizobia, and legumes will evolve cell-surface pattern-recognition receptors (PRRs)^53^,^55^. However, little is known about whether nodule development and senescence are directly associated with the plant immunity defence.

CEBiP and CERK1, two PRRs of chitin, were differentially expressed and down- regulated in nodule development, except that CEBiP was up-regulated in Group 8 (Fig. 5). Analyses of the other DEGs in nodule development showed that Ca+ signalling, MAPK cascade, hypersensitive response, and ubiquitin-mediated proteolysis are associated with nodule development (Fig. 5). Interestingly, most of the selected KEGG gene sets in this pathway were differentially expressed between different developmental periods of soybean (Fig. 5), indicating the initiation or termination of a series of plant immunity defence processes, which might be associated with changes in nodule activity and rhizobia differentiation. These data uncovered some important genes in the tightly regulated soybean nodule nitrogen fixation process that coordinate nodule development with the host soybean immunity defence. However, the mechanism of this co-regulation remains to be determined.

Previous studies have shown that cysteine proteases and cystatins are actively transcribed during nodule development and senescence^11^, and inhibition of some cysteine proteases delay nodule senescence^56^,^57^. Cystatin genes, nature inhibitors of cysteine proteases, are very important in nodule symbiosis and 7 soybean cystatins play different roles in nodulation, nodule development and senescence^58^. In this report, 6 cystatins were identified from our RNA-Seq (Table S5), among them, two cystatins (Glyma18g12240 and Glyma18g00690) were not in the seven cystatins^11^. Glyma18g12240 was significantly increased in roots at 0.5 h of post inoculation and during nodule developments^58^ and different expressed in 6 Groups (Table S5), indicating that Glyma18g12240 play roles in nodulation and nodule development. The symbiotic function of Glyma18g12240 was studied and the results showed that it really promotes nodulation in *L. japonicus* and performs an essential role in delaying nodule senescence (Fig. 7). The exact regulation mechanism of Glyma18g12240 is important and worthy of exploration in further studies.

In this study, we investigated five important developmental stages of soybean by RNA-Seq and identified a large number of nodule-associated DEGs, including soybean symbiotic nitrogen fixation-related proteins, cysteine proteases, cystatins, cysteine-rich proteins and other regulatory proteins. Some important DEGs were also involved in plant immunity defence during nodule development. The DEGs uncovered in this study and their analyses shed new light on nodule development and nitrogen fixation and provide molecular material for further investigations of the mechanisms of nitrogen fixation in different soybean developmental stages.

## Materials and Methods

### Plant Materials and Growth Conditions

Seeds of Soybean Tian long No.1 (Oil Crops Research Institute CAAS in China) were surface-sterilized and grown in pots filled with sterilized vermiculite and perlite (1:1) with half-strength B&D medium in a chamber with a 16/8 h day/night cycle at 28 °C for 4–5 days before inoculation with rhizobium strain *113-2* (Oil Crops Research Institute CAAS in China). After inoculation, the plants were kept under the same growth conditions. The soybean growth conditions and roots were photographed at five developmental stages: branching, flowering, fruiting, pod and harvest stages.

Samples for RNA isolation were collected from soybean nodules at five developmental stages: branching stage (three or four nodes on the main stem with fully developed leaves beginning with the unifoliolate leaves, 11–14 days after inoculation), flowering stage (28–32 days after inoculation), fruiting stage (Pod 5 mm–1 cm long at one of the four uppermost nodes on the main stem with a fully developed leaf, 39–46 days after inoculation), pod stage (Seeds 1/8 inch long in a pod to pod containing a green seed that fills the pod cavity at one of the four uppermost nodes on the main stem with a fully developed leaf, 53–66 days after inoculation), and harvest stage (ninety-five percent of the pods have reached their mature pod colour, 80–88 days after inoculation). Nodules from different stages were separately collected with three biological replicates and then mixed at a ratio of 1:1:1 for sequencing.

### RNA sample preparation and cDNA library construction

Total RNA was extracted using Trizol reagent (Invitrogen, USA) and quantified using an Epoch Multi-Volume Spectrophotometer system, followed by purification with an RNeasy plant mini kit (QIAGEN, Germany) to remove potential genomic DNA. RNA quality and concentration were evaluated using the NanoDrop method and an Agilent 2100 Bioanalyser (Agilent Technologies, Palo Alto, CA, USA). The A260/A280 and A260/A230 ratios of all of the RNA samples that were used in this study were above 2.0.

An equal amount of total RNA from each sample was pooled for RNA-Seq to obtain a comprehensive range of transcripts. Possible DNA contamination was removed from the total RNA samples using DNase I. The mRNA was enriched using the oligo (dT) magnetic beads and then mixed with fragmentation buffer and fragmented into short fragments (approximately 200 bp). First-strand cDNAs were synthesized using random hexamer primers. Buffer, dNTPs, RNaseH, and DNA polymerase I were added to synthesize the second strand. Double-stranded cDNAs were purified using magnetic beads. The fragments were enriched by PCR amplification after ligated sequencing adaptors. The sample library was qualified and quantified using an Agilent 2100 Bioanalyser and ABI Step OnePlus Real-Time PCR System and then subjected to sequencing on an Illumina HiSeq 2000.

### Clean reads library formation

The “raw reads” included some adaptor sequences and/or low-quality reads; therefore, data filtering was carried out to transform “raw reads” into high-quality (clean) reads. The procedure included the following steps: 1. Reads with adaptor sequences were removed. 2. Reads in which the percentage of unknown bases was greater than 10% were removed. 3. Low-quality reads in which the percentage of low-quality bases (bases with a quality value ≤5) was greater than 50% in a read were removed. The clean reads were then mapped (ftp://ftp.jgi-psf.org/pub/compgen/phytozome/v9.0/Gmax/annotation/Gmax_189_transcript.fa.gz and ftp://ftp.jgi-psf.org/pub/compgen/phytozome/v9.0/Gmax/assembly/Gmax_189.fa.gz) using SOAP aligner/SOAP2^59^. No more than two mismatches were permitted in the alignment. Details of the mapping result (mapping to reference genes and the genome) are shown in Supplemental Table S2.

### Screening of DEGs

The fold change in gene expression in combination with the FDR was used to distinguish differentially expressed genes between samples because of the significance of digital gene expression profiles^60^. Our analysis used an FDR ≤0.001 and an absolute value of log_2_ ratio ≥1 as thresholds to judge the significance of gene expression differences for the following ten Groups. Group 1: A comparison of nodules at the branching stage and flowering stage; Group 2: A comparison of nodules at the branching stage and fruiting stage; Group 3: A comparison of nodules at the branching stage and pod stage; Group 4: A comparison of nodules at the branching stage and harvest stage; Group 5: A comparison of nodules at the flowering stage and fruiting stage; Group 6: A comparison of nodules at the flowering stage and pod stage; Group 7: A comparison of nodules at the flowering stage and harvest stage; Group 8: A comparison of nodules at the fruiting stage and pod stage; Group 9: A comparison of nodules at the fruiting stage and harvest stage; Group 10: A comparison of nodules at the pod stage and harvest stage. Data of the DEGs in the ten Groups between the five nodule samples are provided in Supplemental Table S3.

### Gene Ontology functional and KEGG pathway analyses of DEGs

Gene Ontology functional and KEGG pathway analyses of DEGs usually help to understand genes’ biological functions. The method of GO functional enrichment analysis first maps all DEGs to terms in the GO database (http://www.geneontology.org/), calculates the gene numbers for every term, and then finds significantly enriched GO terms in DEGs using a hypergeometric test. The calculating formula is:


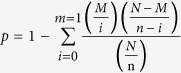


In which N is the total number of genes with GO annotation, n is the number of DEGs in N, M is the total number of genes annotated with the particular GO term, and m is the number of DEGs in M. Our analysis used a Bonferroni-corrected P-value ≤ 0.05 as the threshold to define significantly enriched GO terms in DEGs. Two software programmes, Blast2GO and WEGO, were used in the Gene Ontology functional classification (WEGO) analysis. Blast2GO, which we used to obtain the GO annotation of DEGs, is a widely recognized GO annotation software. After obtaining the GO annotation for DEGs, WEGO software was used for GO functional classification for DEGs and to determine the distribution of gene functions of the species at the macro level.

KEGG is the major public pathway-related database (http://www.kegg.jp/kegg/kegg1.html). The calculating formula for the pathway enrichment analysis was the same as that for the GO analysis. Here, N is the number of all genes with that KEGG annotation, n is the number of DEGs in N, M is the number of all genes annotated to specific pathways, and m is the number of DEGs in M. In addition to the list of the most meaningful pathways, KEGG pathway analysis supplies detailed pathway information by clicking the hyperlinks on the pathways.

### Quantitative real-time PCR (qPCR)

RNA samples were treated with DNase I (Takara) and reverse-transcribed using a Prime Script RT reagent Kit (Perfect Real Time) with gDNA Eraser (Takara Bio, Inc) and oligo (dT) as the primer. Using a Mini Opticon real-time PCR system (Bio-Rad IQ5), cDNA from the reverse transcription of approximately 1 μg of RNA was used as the template for qPCR using the primer sets listed in Supplemental Table S7 and cycling conditions of 30 s at 95 °C followed by 40 cycles of 5 s at 95 °C, 15 s at 60 °C and 12 s at 72 °C, and a final 5 s at 72 °C. The QACT and/or polyubiquitin transcript were used as internal controls. Sample cycle threshold (CT) values were standardized for each template using the reference gene as a control, and the 2^−ΔΔCT^ method^61^ was used to analyse the relative changes in gene expression from the qPCR experiments. Three biological replica samples and three replicate reactions per sample were used to ensure statistical credibility.

### Overexpression of *Glyma18g12240* in *L. japonicus* by hairy root transformation

The full-length coding sequence of Glyma18g12240 was cloned into the *KpnI/BamHI* site of pU1301 (Primer-F: 5′-GGTACCATGGCAATGATAGGAGGC-3′; Primer-R: 5′-GGATCCTTAGCTGGGTGCAGCATAAG-3′), generating pMUb: Glyma18g12240. *A. rhizogenes* strain LBA1334 cells carrying pMUb: Glyma18g12240 were used to induce hairy root formation in Wild-type *L. japonicus* ‘MG-20’ using an *A. rhizogenes*-mediated procedure as described previously^47^,^48^. Briefly, seedlings were cut at the base of the hypocotyls, placed in a suspension of *A. rhizogenes* LBA1334 cells containing plasmids for 30 min, and then transferred onto agar plates of Murashige and Skoog (MS; Sigma) medium containing 1.5% (w/v) Suc and co-cultivated for 5 d. The plants were then transferred onto agar plates containing 250 mg/ml of cefotaxime and grown until hairy roots (a few cm long) developed from the base of the hypocotyls (approximately two weeks). Each hairy root was labelled, and a short root tip (1 to 3 mm long) was removed to test for GUS activity in staining solution overnight at 37 °C in the dark. Hairy roots with GUS-positive tips were preserved and allowed to continue to grow. Each seedling was allowed to have 1 to 2 transgenic hairy roots. Plants with transgenic hairy roots were transferred to pots filled with vermiculite and sand (1:1) with one-half-strength Broughton &Dilworth (B&D) medium^62^ and grown in a chamber in a 16-/8-h day/night cycle at 22 °C. After a week of adaptation, the plants were inoculated with *Mesorhizobium loti* strain MAFF303099 and grown in the same medium without ammonium nitrate. The nodulation phenotypes of transgenic hairy roots were scored 49 days after inoculation with *M. loti*. Transgenic hairy roots expressing the empty vector (pU1301) were used as controls.

### Paraffin-embedded slides analysis

We harvested both control and Glyma18g12240-OX nodules at 49 dpi (days post inoculation). Then, 20 randomly selected larger nodules were collected in the zone near the base of the root for paraffin-embedded slides analysis (both for control and Glyma18g12240-OX). Horizontal sections through nodules were fixed in FAA buffer, washed, dehydrated, and embedded in paraffin. We prepared small sections using a microtome and mounted these on glass slides. The slides were stained with toluidine blue and observed using a light microscope. Then, the pictures were processed by Pannoramic viewer.

## Additional Information

**How to cite this article:** Yuan, S. L. *et al*. RNA-Seq analysis of nodule development at five different developmental stages of soybean (*Glycine max*) inoculated with *Bradyrhizobium japonicum* strain 113-2. *Sci. Rep.* **7**, 42248; doi: 10.1038/srep42248 (2017).

**Publisher's note:** Springer Nature remains neutral with regard to jurisdictional claims in published maps and institutional affiliations.

## Supplementary Material

Supplementary Files-1

Supplementary Files-2
