# Supplementary material for: RNA-Seq analysis of nodule development at five different developmental stages of soybean (Glycine max) inoculated with Bradyrhizobium japonicum strain 113-2
Source: Sci Rep. 2017 Feb 7;7:42248. doi: 10.1038/srep42248 (PMC5294573; doi:10.1038/srep42248)
Supplement: Supplementary Files-1 [file srep42248-s1.pdf]

RNA-Seq analysis of nodule development at five different developmental stages of soybean (*Glycine max*) inoculated with *Bradyrhizobium japonicum* strain 113-2

Song L Yuan[1,2], Rong Li[1,2], Hai F Chen[1,2], Chan J Zhang[1,2], Li M Chen[1,2], Qing N Hao[1,2], Shui L Chen[1,2], Zhi H Shan[1,2], Zhong L Yang[1,2], Xiao J Zhang[1,2], De Z Qiu[1,2] and Xin A Zhou[1,2*]

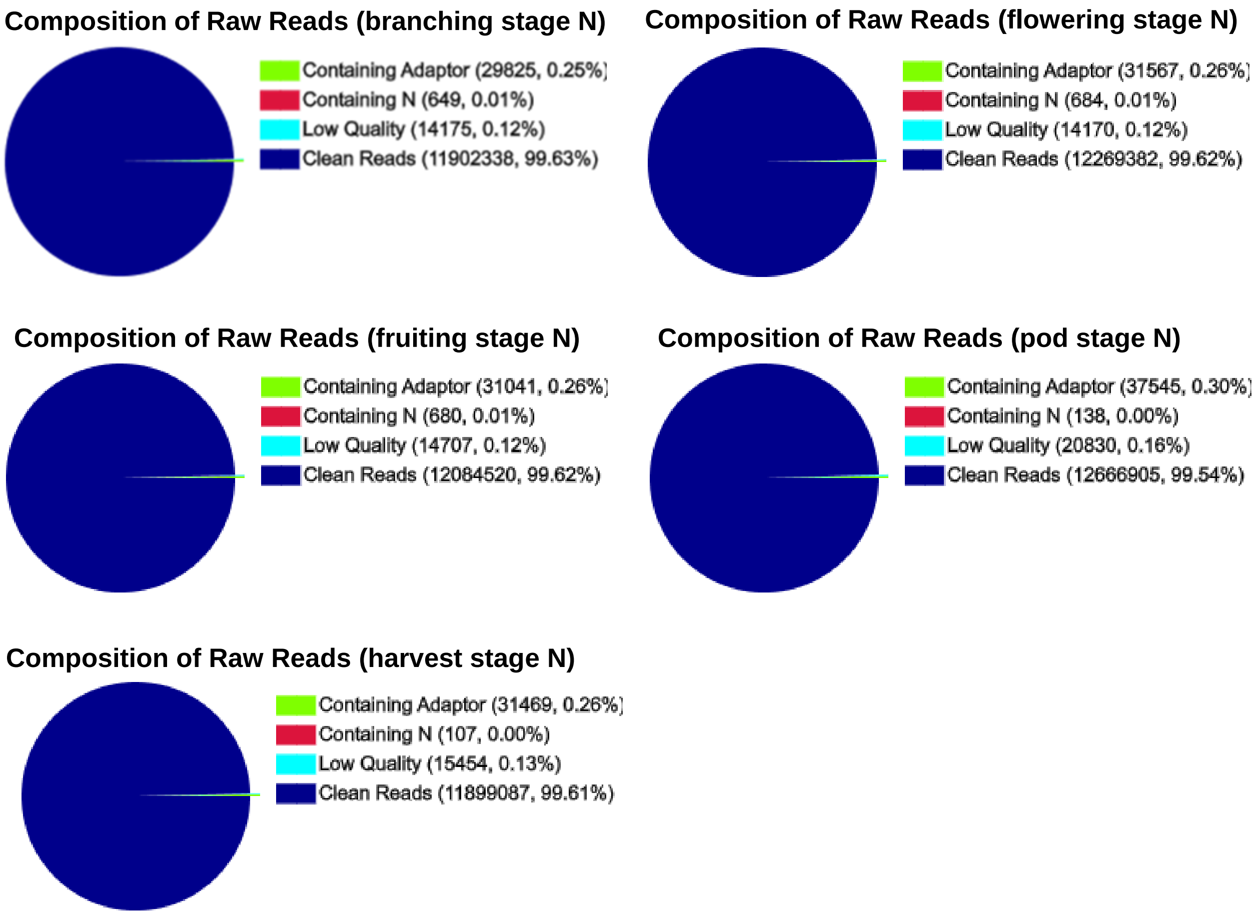

Figure S1: Representative results of the classification of raw reads for nodules samples at five developmental periods.

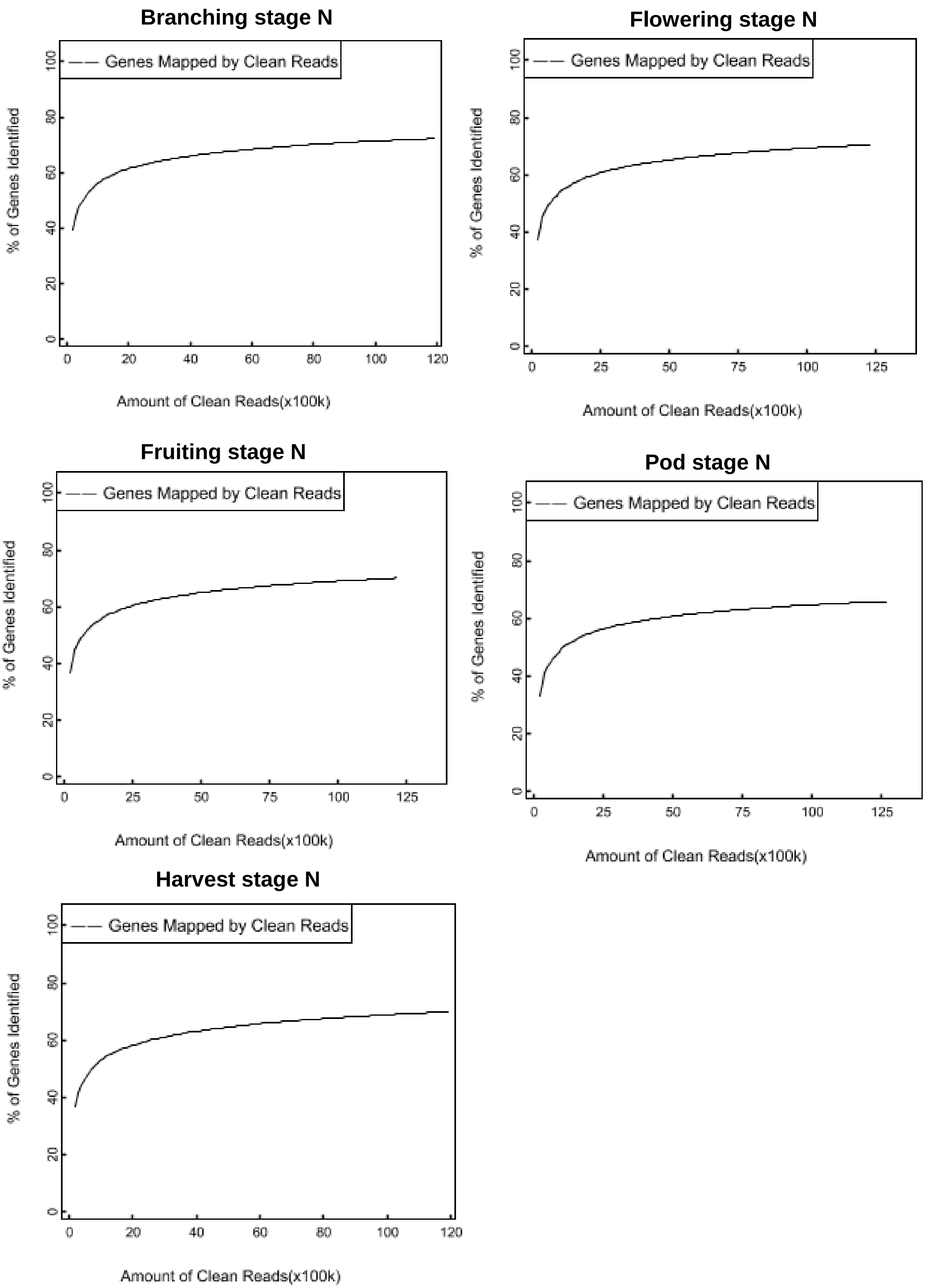

Figure S2: Representative results of sequencing saturation analysis for nodules sampled at five developmental periods.

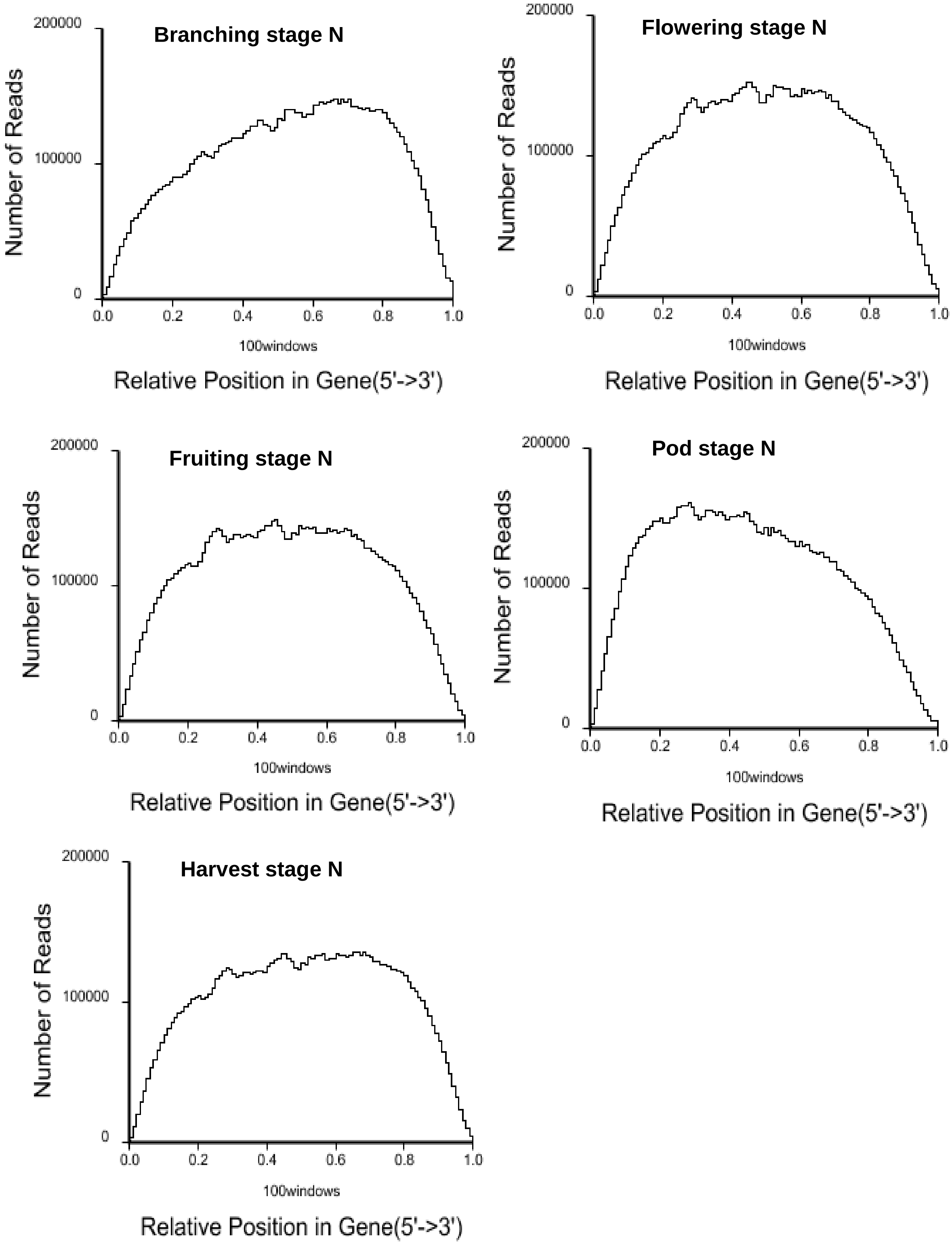

Figure S3: Representative results of distributions of reads on reference genes for nodules sampled at five developmental periods.

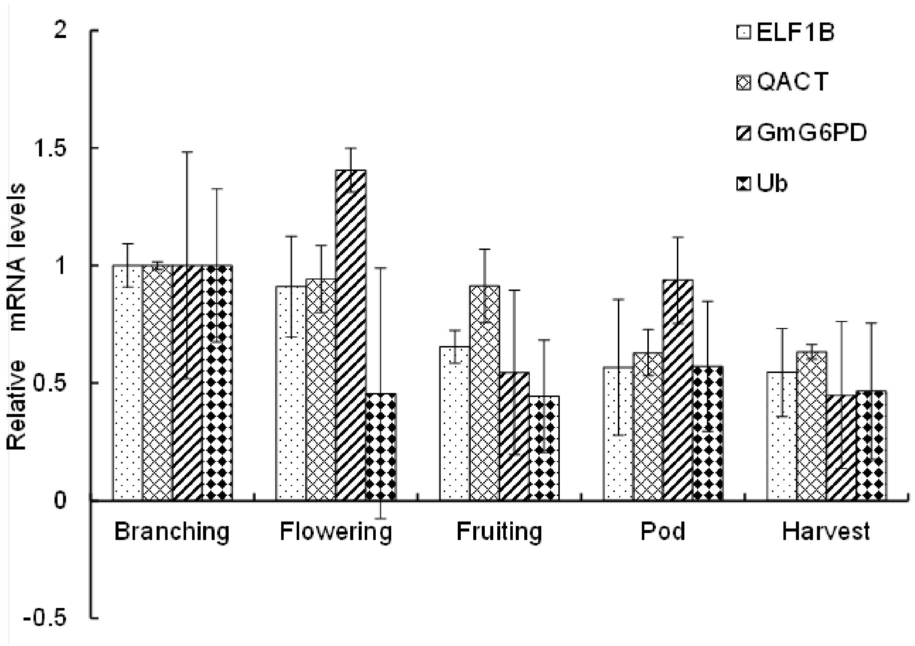

Figure S4: The stability assay of four references genes at five developmental stages of soybean.

Table S1: Analysis of nodulation phenotypic in soybean inoculated with rhizobium strains113-2.

	**mean value ±SE**	
	Number of nodules per plant	Dry Weight per nodule(mg)
Branching	13. 125±1. 548(N=24)	–
Flowering	42. 375±1. 908(N=24)	1. 366±0. 050
Fruiting	51. 520±5. 337(N=25)	2. 133±0. 146
Pod	37. 400±1. 830(N=25)	1. 071±0. 044
harvest	14. 500±1. 558(N=15)	1. 697±0. 26
P Value (Branching/Flowering)	0. 445987055	–
P Value (Branching/Fruiting)	0. 921888575	–
P Value (Branching/Pod)	0. 671463359	–
P Value (Branching/harvest)	–	–
P Value (Flowering/Fruiting)	0. 136749853	–
P Value (Flowering/Pod)	0. 103619795	–
P Value (Flowering/harvest)	–	–
P Value (Fruiting/Pod)	0. 014539473	–
P Value (Fruiting/harvest)	–	–
P Value (Pod/harvest)	–	–

Table S2: Summary of the mapping result (mapping to reference genes and the genome).

	branching stage N	flowering stage N	fruiting stage N	pod stage N	harvest stage N
Map to Gene					
total mapped reads	10362371(87.06%)	10945168(89.21%)	10750603(88.96%)	11050021(87.24%)	10163957(85.42%)
perfect match reads	8894432(74.73%)	9371580(76.38%)	9140987(75.64%)	9296356(73.39%)	8646303(72.66%)
unique match reads	8881615(74.62%)	9424020(76.81%)	9268099(76.69%)	9673766(76.37%)	8811312(74.05%)
multi-position match	1480756(12.44%)	1521148(12.4%)	1482504(12.27%)	1376255(10.86%)	1352645(11.37%)
Map to Genome					
total mapped reads	10183173(85.56%)	10490977(85.51%)	10287336(85.13%)	10635747(83.96%)	9718196(81.67%)
perfect match reads	8531889(71.68%)	8736557(71.21%)	8498873(70.33%)	8706798(68.74%)	8031614(67.5%)
unique match reads	8644266(72.63%)	8991605(73.28%)	8856144(73.29%)	9300692(73.43%)	8417920(70.74%)
multi-position match	1538907(12.93%)	1499372(12.22%)	1431192(11.84%)	1335055(10.54%)	1300276(10.93%)

Table S4: Lists of DEGs with over 8-fold changes in the plant–pathogen interaction KEGG pathway.

Gene sets	Gene	Groups
**MKK1/2**	Glyma15g18860	Group 1,4
**PR1**	Glyma17g07350	Group 1
	Glyma13g32560	Group 1
	Glyma15g06830	Group 5,8
**CEBiP**	Glyma06g11820	Group 2,4
**RPS5**	Glyma15g02870	Group 3
	Glyma18g14810	Group 3
	Glyma08g16041	Group 3,6
	Glyma19g07680	Group 3
	Glyma18g51546	Group 6,8,10
	Glyma16g34030	Group 6,8,10
	Glyma15g39531	Group 6,8
	Glyma08g12560	Group 10
**PBS1**	Glyma06g46971	Group 5
**WRKY2209**	Glyma19g40470	Group 7,9
	Glyma16g03570	Group 7,9

**RIN4**	Glyma09g01151	Group 7,10
**EFR**	Glyma07g17910	Group 10
**RPS2**	Glyma18g51546	Group 10
	Glyma16g34030	Group 10
	Glyma08g12560	Group 10
	Glyma02g04750	Group 10
	Glyma17g21200	Group 10

Table S5: Cysteine proteinases, cystatins and cysteine-rich proteins identified in nodule development and senescence using RNA-Seq.

		**cysteine-rich genes**			
**Gene**	**Groups**	**Annotation**	**Gene**	**Groups**	**Annotation**
Glyma10g35100	Group 3-4,6-9	cysteine proteinase	Glyma09g40681	Group 1-4,6,8	protein kinases
Glyma12g04340	Group 7,9	cysteine proteinase	Glyma10g39910	Group 4,10	protein kinases
Glyma17g18440	Group 1-3	cysteine proteinase	Glyma11g32301	Group 3,7,8,10	protein kinases
Glyma11g12130	Group 2-4,7	cysteine proteinase	Glyma11g32500	Group 3,6,8,10	protein kinases
Glyma14g09440	Group 3-4,6,8	cysteine proteinase	Glyma13g25800	Group 3,6,8,10	protein kinases
Glyma17g35720	Group 3-4,6-9	cysteine proteinase	Glyma09g27782	Group 1,2,4,6,8,10	protein kinases
Glyma04g03020	Group 3,10	cysteine proteinase	Glyma20g27780	Group 1,2,4,6,8,10	protein kinases
Glyma15g19580	Group 3,6	cysteine proteinase	Glyma08g39150	Group 4	protein kinases
Glyma14g40670	Group 1-7,10	cysteine proteinase	Glyma07g24010	Group 1,2,4,10	protein kinases
Glyma09g08100	Group 3-4,6-9	cysteine proteinase	Glyma20g27460	Group 1,2,4	protein kinases
Glyma04g36470	Group 1-4,6-9	cysteine proteinase	Glyma18g20470	Group 1,2,4	protein kinases
Glyma06g18390	Group 1,3-9	cysteine proteinase	Glyma20g27720	Group 2	protein kinases
Glyma18g12240	Group 1-2,4,6,8,10	cystatin	Glyma08g10030	Group 3	protein kinases
Glyma05g28250	Group 1-2,4	cystatin	Glyma13g25811	Group 1-4	protein kinases
Glyma15g36180	Group 3	cystatin	Glyma02g04210	Group 1,2,4,8,10	protein kinases
Glyma15g12211	Group 2-4,6	cystatin	Glyma11g32520	Group 3-7,9	protein kinases
Glyma14g04250	Group 1-5,7,10	cystatin	Glyma20g27580	Group 4,7	protein kinases
Glyma18g00690	Group 1-5	cystatin	Glyma18g53186	Group 1-5,7,9,10	protein kinases
Glyma10g26900	Group 1-5,7-10	secretory protein	Glyma18g45175	Group 1-4	protein kinases
Glyma13g01250	Group 1-4,7,9,10	secretory protein	Glyma04g15456	Group 1-4	protein kinases
Glyma05g34990	Group 1-5,7-8	secretory protein	Glyma11g31995	Group 2-7,9,10	protein kinases
Glyma18g04610	Group 1-4,6-7,9-10	secretory protein	Glyma20g27660	Group 1-4,7,9,10	protein kinases
Glyma08g04730	Group 2,4,5,8,10	secretory protein	Glyma20g27670	Group 1-4,7,9,10	protein kinases
Glyma20g34230	Group 2,5	secretory protein	Glyma20g27690	Group 1-4,7,9,10	protein kinases
Glyma19g35130	Group 2-5,7	secretory protein	Glyma20g27790	Group 1-4	protein kinases
Glyma14g39440	Group 3,4,7,9,10	secretory protein	Glyma11g32600	Group 2-10	protein kinases
Glyma13g38170	Group 2-7,9	secretory protein	Glyma01g45160	Group 3-4,6-10	protein kinases
Glyma14g14750	Group 2-8,10	polycomb protein	Glyma11g32041	Group 2-4,6-10	protein kinases
Glyma17g31399	Group 3,10	polycomb protein	Glyma10g39961	Group 1,3-10	protein kinases
Glyma10g39870	Group 1-4,7,9,10	protein kinases	Glyma18g05260	Group 1-9	protein kinases

Glyma10g15170	Group 1-4,8,10	protein kinases	Glyma11g32590	Group 1-4,6-9	protein kinases

Table S6: Symbiotic nitrogen fixation - related genes identified in nodule development and senescence using RNA-Seq.

Symbiotic nitrogen fixation - related genes			
**Gene**	**Groups**	**Annotation**	**Potential symbiotic function**
Glyma01g03470	Group 1-5,7,8,10	*Enod 40*	Nodule development
Glyma10g23790	Group 3,6,8,10	*Nodulin 35*	Assimilation of the fixed nitrogen
Glyma16g04750	Group 1-4,7,9,10	*ApyraseGS52*	Nodulation
Glyma02g06680	Group 10	*Calmodulin-like*	Nodulation
Glyma19g45310	Group 3,8,10	*MtDMI1-like*	Nodulation
Glyma09g33510	Group 3,6,8,10	*MtDMI2-like*	Nodulation
Glyma16g04410	Group 1-8,10	*MtERN1-like*	Nodulation
Glyma19g29000	Group 1-4,6,7	*MtERN1-like*	Nodulation
Glyma01g35255	Group 2-4,6,8,10	*MtIPD3-like*	Nodulation
Glyma09g34695	Group 3-4,6-8,10	*MtIPD3-like*	Nodulation
Glyma10g33851	Group 6,8,10	*MtLIN-like*	Nodulation
Glyma02g43860	Group 1-9	*MtLYK3-like*	Nodulation
Glyma02g43850	Group 1-6,9,10	*MtLYK3-like*	Nodulation
Glyma01g38550	Group 1-3,7,9-10	*MtLYR3-like*	Nitrate transporter
Glyma04g00210	Group 3-4	*MtNIN-like*	Nodulation
Glyma07g04430	Group 4,7,9	*MtNSP1-like*	Nodulation
Glyma06g11610	Group 2,4,5,7-9	*MtNSP2-like*	Nodulation
Glyma10g07190	Group 1-8,10	*MtRRP1-like*	Nodulation
Glyma03g33850	Group 7	*MtSKL1-like*	Nodulation
Glyma12g04390	Group 1-6,10	*MtSUNN-like*	Regulates nodule number
Glyma20g38950	Group 3,6,8	*GmN56*	Nitrogen fixation
Glyma13g12484	Group 6	*GmN56*	Nitrogen fixation
Glyma19g29880	Group 1-7,9,10	*GmN56*	Nitrogen fixation
Glyma05g08400	Group 1-4,7-9	*GmENOD93*	Nodule development
Glyma17g12610	Group 1,2,6,8,9	*GmENOD93*	Nodule development

Table S7: Primers for genes selected for semi-quantitative RT-PCR and qPCR analysis

**Gene**	**Forward primer**	**Reverse primer**
Ubiquitin(soybean)	5'-GCTCGTTGTGTAATGTTGG-3'	5'-CGTAGGTGGGATATTAGAGT-3'
QACT(soybean)	5'-ATCTTGACTGAGCGTGGTTATTCC-3'	5'-GCTGGTCCTGGCTGTCTCC-3'
ELF1B(soybean)	5'-GTTGAAAAGCCAGGGGACA-3'	5'-TCTTACCCCTTGAGCGTGG-3'

Gmg6po(soybean)	5'-ACTCCTTGATACCGTTGTCCAT-3'	5'-GTTTGTTATCCGCCTACAGCCT-3'
Glyma06g11730	5'-GTCACGTGTGAACTACCAGGA-3'	5'-AGCTATATGGCCTAATCTCTGGA-3'
Glyma20g34140	5'-ACGGCTTGATAAGGCTCCAAA-3'	5'-AGGGTTACACAATAACTCGCC -3'
Glyma06g18390	5'-TCTCAGATTCAATGCCAGTGT-3'	5'-TGAAACTCTTGGCCATAGAAAGG-3'
Glyma05g26800	5'-TGTGAATCCCCCTTCTTCAGC-3'	5'-GACAACACAAGTTCTGCAGTT-3'
Glyma14g07870	5'-GCCTCACTGGTATGTGACTGT-3'	5'-TGGACGTGAACAAAGAAGAGGT-3'
Glyma15g13290	5'-TTATTCACGCAAGCTGGGTTC-3'	5'-GGAGGTCAAATAAACAAAGGAGC-3'
Glyma03g29670	5'-ACTGTGGCATGTGGAGGAAC-3'	5'-TGTCAAACCACTTTTCATGGCTC-3'
Glyma08g06690	5'-TGGTCCTTTTCTTTCAGCACCT-3'	5'-CAGCCCAAAATAGCGAGGGA-3'
Glyma10g34280 (Lb)	5'-ACAATAAAGGAAGCTGTTGGCGG-3'	5'-TTACACTTTACGGCAATGCAG-3'
NIN (*L.japonicus*)	5'-AACTCACTGGAAACAGGTGCTTTC-3'	5'-CTATTGCGGAATGTATTAGCTAGA-3'
Enod40 (*L.japonicus*)	5'-CAAAACTCGTTATGTTGCGG-3'	5'-CACCTC AAAGGA AGAAGA ACA-3'
Lb (*L.japonicus*)	5'-CTCCAAGCCCATGCTGAAAA-3'	5'-TGGCATCTGCAAGTGTCACTTC-3'
Ubiquitin (*L.japonicus*)	5'-TTCACCTTGTGCTCCGTCTTC-3'	5'-AACAACCAGCACACACAGACAATC-3'
Glyma18g12240	5'- GGTACCATGGCAATGATAGGAGGC -3'	5'- GGATCCTTAGCTGGGTGCAGCATAAG - 3'
